# Unraveling Biofilm‐Forming Uropathogens: Isolation and Antimicrobial Resistance Patterns at Nepal Police Hospital, Kathmandu

**DOI:** 10.1155/cjid/6625304

**Published:** 2026-04-26

**Authors:** Niroj Man Amatya, Samena Shrestha, Nikita Syangtan Tamang, Muna Bista, Shruti Gautam, Jayandra Shrestha, Samriddhi Karki

**Affiliations:** ^1^ Department of Medical Microbiology, Nobel College, Kathmandu, Nepal; ^2^ Pathology Department, Nepal Police Hospital, Maharajgunj, Kathmandu, Nepal

**Keywords:** antimicrobial challenge, biofilm, MDR *Klebsiella pneumoniae*, UTI

## Abstract

**Background:**

Urinary Tract Infections (UTIs) are common bacterial infections with growing treatment challenges due to the rise of antimicrobial resistance. Additionally, the characteristics of the pathogens responsible for UTIs are changing, primarily due to the emergence of biofilms. Biofilms, which are structured microbial communities, pose a significant public health threat because of their inherent resistance to antimicrobial treatments. Hence, this cross‐sectional study aimed to isolate and characterize uropathogenic bacteria capable of forming biofilm and exhibiting antimicrobial resistance among patients seeking microbiology laboratory services at the Nepal Police Hospital.

**Methods:**

Uropathogens were isolated from midstream urine samples using CLED, Blood, and MacConkey agar. Standard microbiological techniques were employed for identification of pathogen. Antimicrobial susceptibility testing was conducted using the Kirby–Bauer disc diffusion method, and biofilm formation was assessed using the microtiter plate method.

**Results:**

Of the 2081 samples analyzed, significant bacterial growth was observed in 184 (8.84%) of the samples. The predominant pathogens were *Escherichia coli* (39.7%), followed by *Klebsiella pneumoniae* (26.6%) and *Pseudomonas aeruginosa* (7.8%). Amikacin, levofloxacin, and tigecycline were the most effective antibiotics. Among the isolates, 32 (17.39%) were confirmed as MDR. Biofilm production was confirmed in six isolates (3.26%), with two *Enterococcus faecalis* and one *Klebsiella oxytoca* identified as strong biofilm producers, while two *Klebsiella pneumoniae* and one *Acinetobacter* spp. exhibited weak biofilm production. Statistical analysis showed no significant correlation between antibiotic resistance and biofilm production (*p* > 0.05).

**Conclusion:**

Biofilm‐forming uropathogens present substantial challenges in UTI treatment. Importantly, our study did not find a correlation between antibiotic resistance and biofilm production, suggesting that these traits may be independent or influenced by different pathogenic mechanisms.

## 1. Introduction

Urinary tract infection (UTI) is frequently associated with structural or neurological abnormalities in the urinary tract, including the urethra, urinary bladder, prostate, and kidney. These infections present with a wide spectrum of clinical manifestations, ranging from asymptomatic bacteriuria and subclinical infections to severe and potentially life‐threatening conditions such as urosepsis. It is the most common microbial infection, primarily caused by *Escherichia coli*, *Klebsiella pneumoniae*, and other Gram‐positive bacteria, including fungi occurring in both community and healthcare settings [1, 2]. The presence of clinical complications, high prevalence rates, and the mounting number of antibiotic resistances in these uropathogens make UTIs a matter of considerable clinical practical interest.

Over the last decade, significant variations in the incidence of UTIs have been observed across different sociodemographic groups, nations, regions, sexes, and age groups. Although comprehensive global data remain limited, recent spatiotemporal analyses have revealed a substantial rise in UTI cases; an incidence of 60.4%, from 252.25 million in 1990 to 404.61 million in 2019. Similarly, UTI‐related mortality surged by 140.18%, rising from 98,590 deaths in 1990 to 236,790 deaths in 2019 [3]. With this rising prevalence rate, the issue of antibiotic resistance among commonly used antibiotics has also escalated, alongside resistance to carbapenem antibiotics [4].

Additionally, the potential for biofilm formation by uropathogens is another important factor that shields bacteria from both the immune system and antibiotics [5]. The havoc created by these multidrug‐resistant (MDR) uropathogens can substantially impact both individuals and healthcare systems. These consequences encompass heightened healthcare expenses, suboptimal health outcomes, restricted treatment options, an elevated risk of transmission, increased rates of morbidity and mortality, and reduced quality of life [6–8].

Therefore, studying uropathogenic biofilm producers and their antibiotic resistance profiles provides insight into recent trends in antibiotic resistance and aims to improve empirical guidance for alternative antibiotic treatment strategies.

## 2. Materials and Methods

A cross‐sectional study was conducted among patients with suspected UTI seeking microbiological investigation across all age groups at Nepal Police Hospital, Kathmandu, starting from May 28, 2023, to August 30, 2023. From an aseptically collected mid‐stream urine sample only, a 1 μL sample was inoculated on CLED agar and incubated at 37°C for 24 h. A bacterial count greater than 10^5^ CFU/mL was considered the threshold value for significant bacteriuria [9].

The isolated microbes were identified using standard microbiological tests, including Gram reaction, colony morphology, and various biochemical tests. For Gram‐negative bacteria, the tests were catalase, oxidase, sulphide indole motility test, citrate test, triple sugar iron test, and urease test. For Gram‐positive bacteria, the tests were catalase, coagulase, and bile‐esculin test [10]. Antibiotic susceptibility testing was carried out using the Kirby–Bauer disc diffusion method on Mueller Hinton agar, as recommended by the Clinical Laboratory Standards Institute [11]. The antibiotics amikacin, amoxicillin, cephalexin, ceftriaxone, cefixime, levofloxacin, cotrimoxazole, nitrofurantoin, and norfloxacin were tested against both Gram‐positive and Gram‐negative bacterial isolates. Additionally, amoxicillin‐clavulanate, ceftazidime, cefepime, piperacillin, piperacillin‐tazobactam, imipenem, meropenem, and tigecycline were tested exclusively against Gram‐negative bacteria, whereas doxycycline and vancomycin were tested only for Gram‐positive bacterial isolates. Isolates showing resistance to at least one agent in three or more antimicrobial categories were considered MDR [12].

Biofilm formation was assessed using a microtiter plate‐based assay. The final bacterial inoculum (5 × 10^5^ CFU/mL) in 200 μL Mueller–Hinton Broth supplemented with 1% glucose was added to a flat‐bottom 96‐well polystyrene microtiter plate and incubated aerobically at 37°C for 48 h. The plate was then thoroughly washed three times with phosphate‐buffered saline (PBS, pH 7.2), baked at 60°C for 60 min, and stained with 2% crystal violet (150 μL) for 15 min at room temperature. After aspirating the stain and air‐drying, 150 μL ethanol (95%) was added for 30 min at room temperature to detach the fixed cells from the plate. The optical density (OD) of each well was measured (λmax = 570 nm) using an ELISA plate reader (SPECTROstar, BMG LABTECH). The cut‐off value (ODc) was defined as three standard deviations above the mean OD of negative control. The biofilm‐forming ability of isolates was differentiated as nonbiofilm producers (OD ≤ ODc) and biofilm producers, which were further differentiated into weak (ODc < OD ≤ 2 × ODc), moderate (2 × ODc < OD ≤ 4 × ODc), and strong biofilm producers (4 × ODc < OD) [13]. All assays were performed in triplicate. Since biofilm formation and MDR property are both independent and categorical variables, their correlation was statistically evaluated by using chi‐square test. Ethical approval for this study was obtained from the Nobel Institution Research Committee.

All procedures were performed under strict aseptic conditions. *E. coli* ATCC 25922 was used as a control strain. Data were analyzed using SPSS version 23. A *p* value of less than 0.05 was considered significant.

## 3. Results

### 3.1. Patients Characterization

A total of 2081 urine samples were received at the Microbiology Department for culture and susceptibility testing. The minimum and maximum ages of the patients were below 1 year and 95 years, respectively. The mean age was 38.65 years, with a standard deviation of 19.76. The distribution of patients according to age, sex, and sample origin is presented in Table 1.

**TABLE 1:** Demographic information.

Status of patients	Total number	Positive case
Outpatients	629 (30.22%)	47 (25.54%)
Inpatients	1238 (59.50%)	117 (63.59%)
Emergency	214 (10.28%)	20 (10.87%)

**Sex**	**Number**	**Positive case**

Male	959 (46.08%)	78 (42.39%)
Female	1122 (53.91%)	106 (57.61%)

**Age Group**	**Number**	**Positive case**

0–20	291 (13.98%)	31 (16.85%)
20–40	860 (41.32%)	56 (30.43%)
40–60	593 (28.50%)	55 (29.89%)
60 and above	337 (16.20%)	42 (22.85%)

### 3.2. Types of Uropathogens

Of the 2081 urine samples, 184 (8.84%) showed significant bacteriuria, with a predominance of Gram‐negative isolates, notably Enterobacteriaceae. Among all isolates, *Escherichia coli* and *Klebsiella pneumoniae* were the most dominant. The details of uropathogens and their numbers among males and females are presented in Table 2.

**TABLE 2:** Types of uropathogens.

Bacterial isolates	Microbes isolates from		Number (%)
Gram‐negative	Male (%)	Female (%)	
*Escherichia coli*	25 (32.1)	48 (45.3)	73 (39.7)
*Klebsiella pneumoniae*	20 (25.6)	29 (27.4)	49 (26.6)
*Klebsiella oxytoca*	7 (9)	6 (5.7)	13 (7.1)
*Enterobacter* spp.	0	2 (1.9)	2 (1.1)
*Citrobacter* spp.	0	3 (2.8)	3 (1.6)
*Acinetobacter* spp.	3 (3.8)	1 (0.9)	4 (2.2)
*Proteus mirabilis*	5 (6.4)	1 (0.9)	6 (3.3)
*Pseudomonas aeruginosa*	10 (12.8)	4 (3.8)	14 (7.6)
Total (Gram‐negative)	**70**	**94**	**164 (89.13)**

*Gram-positive*			
CONS	1 (1.3)	5 (4.7)	6 (3.3)
*Enterococcus faecalis*	6 (7.7)	4 (3.8)	10 (5.4)
*Enterococcus faecium*	1 (1.3)	2 (1.9)	3 (1.6)
*Staphylococcus aureus*	0	1 (0.9)	1 (0.5)
Total (Gram‐positive)	**8**	**12**	**20 (10.87)**
Total	**78 (42.39)**	**106 (57.61)**	**184**

### 3.3. Antibiotic Susceptibility Pattern of Uropathogens

The antibiotic susceptibility test revealed an increasing trend of resistance in *E. coli* and *Klebsiella* species to commonly used antibiotics, including cotrimoxazole, nitrofurantoin, and cephalosporins. Among the predominant microbes, *E. coli*, *Klebsiella* spp., and *Pseudomonas aeruginosa*, no single organism was found to be fully susceptible to all tested antibiotics. Additionally, the high resistance rates to antipseudomonal antibiotics, such as amikacin, ceftazidime, and imipenem, raise significant public health concerns. The remaining antibiotic resistance rates for Gram‐negative isolates are shown in Table 3.

**TABLE 3:** Antibiotic susceptibility pattern of Gram‐negative isolates.

Antibiotics	Number of resistance isolates (resistance percentage)							
	*Escherichia coli*	*Klebsiella pneumoniae*	*Klebsiella oxytoca*	*Pseudomonas aeruginosa*	*Proteus mirabilis*	*Acinetobacter* spp.	*Citrobacter* spp.	*Enterobacter* spp.
Amikacin	2 (2.7)	16 (32.7)	3 (23.1)	10 (71.4)	0	1 (25)	0	1 (50)
Amoxicillin	44 (60.3)	IR	IR	IR	NT	IR	IR	IR
Cephalexin	19 (26.0)	23 (46.9)	9 (69.2)	IR	NT	IR	IR	IR
Ceftriaxone	16 (21.9)	20 (40.8)	7 (53.8)	IR	0	2 (50)	1 (33.3)	2 (100)
Cefixime	17 (23.3)	24 (49)	8 (61.5)	IR	0	2 (50)	NT	2 (100)
Levofloxacin	15 (20.05)	15 (30.6)	4 (30.8)	10 (71.4)	2 (33.3)	1 (25)	0	2 (100)
Cotrimoxazole	25 (34.2)	22 (44.9)	9 (69.2)	IR	4 (66.7)	1 (25)	2 (66.7)	2 (100)
Nitrofurantoin	3 (4.1)	20 (40.8)	6 (46.2)	IR	IR	IR	2 (66.7)	1 (50)
Norfloxacin	15 (21.1)	16 (34.8)	7 (53.8)	11 (78.6)	2 (33.3)	1 (25)	0	2 (100)
Amoxyclav^∗^	NT	14 (100)	3 (50)	IR	NT	IR	IR	1 (100)
Ceftazidime^∗^	NT	13 (92.9)	3 (50)	9 (100)	NT	NT	NT	1 (100)
Cefepime^∗^	NT	14 (100)	2 (33.3)	8 (88.9)	NT	NT	NT	1 (100)
Piperacillin^∗^	NT	12 (85.7)	4 (66.7)	8 (88.9)	NT	NT	NT	1 (100)
Pip/Tazobactam^∗^	NT	12 (85.7)	3 (50)	6 (66.7)	NT	NT	NT	1 (100)
Imipenem^∗^	NT	8 (57.1)	3 (50)	9 (100)	NT	NT	NT	1 (100)
Meropenem^∗^	NT	8 (57.1)	2 (33.3)	8 (88.9)	NT	NT	NT	1 (100)
Tigecycline	NT	1 (7.1)	2 (40)	IR	IR	NT	NT	0

Abbreviations: IR, Inherently resistant; NT, not tested.

^∗^Second line antibiotics for *Klebsiella* spp. only.

Compared to Gram‐negative isolates, the number of Gram‐positive isolates was scant. Antibiotic resistance in *Enterococcus faecalis* was observed for levofloxacin and doxycycline. The antibiotic resistance profiles of all Gram‐positive isolates are summarized in Table 4.

**TABLE 4:** Antibiotic susceptibility pattern of Gram‐positive isolates.

Antibiotics	Number of isolates (resistance percentage)			
	CONS (6)	*Enterococcus faecium* (3)	*Enterococcus faecalis* (10)	*Staphylococcus aureus* (1)
Amikacin	0	IR	IR	0
Amoxicillin	0	NT	NT	0
Cephalexin	0	IR	IR	1 (100)
Ceftriaxone	0	IR	IR	0
Cefixime	2 (33.3)	IR	IR	NT
Levofloxacin	0	0	6 (60)	NT
Cotrimoxazole	2 (33.3)	IR	IR	0
Doxycycline	0	0	2 (25)	0
Nitrofurantoin	0	0	0	0
Norfloxacin	NT	0	0	0
Vancomycin	NT	0	0	NT

Abbreviations: IR, inherently resistant; NT, not tested.

### 3.4. Distribution of MDR in Bacterial Isolates

**FIGURE 1:**
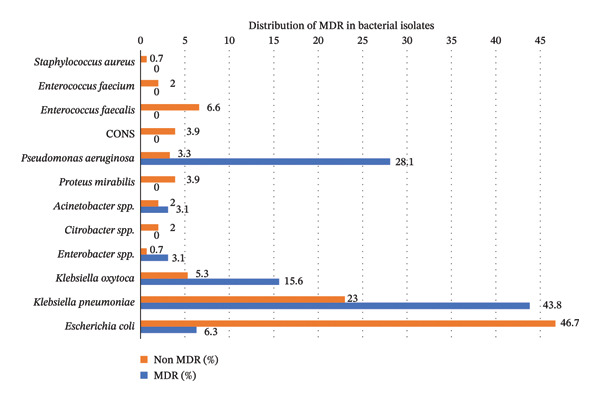
Distribution of MDR and non MDR among uropathogens.

**FIGURE 2:**
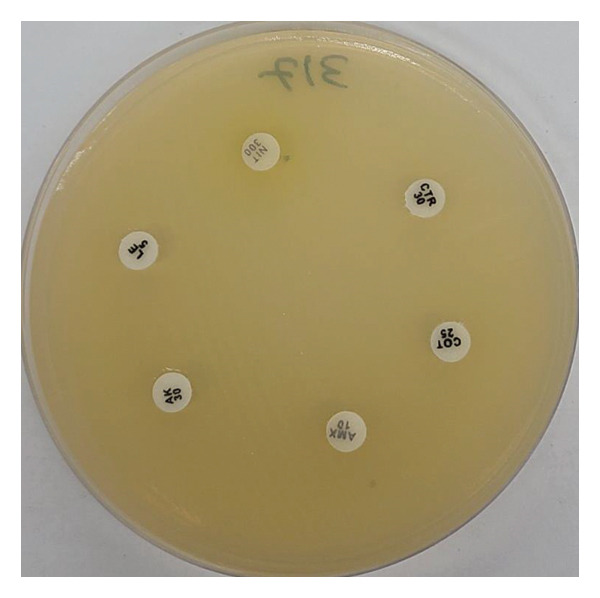
MDR *Klebsiella pneumoniae*.

Of the total isolates, the incidence of MDR was highest in *K*. *pneumoniae* (43.8%), followed by *P. aeruginosa* (28.1%), *K*. *oxytoca* (15.6%), and others (Figure 1). Figure 2 shows the MDR *K. pneumoniae*.

### 3.5. Distribution of Biofilm Producer

**FIGURE 3:**
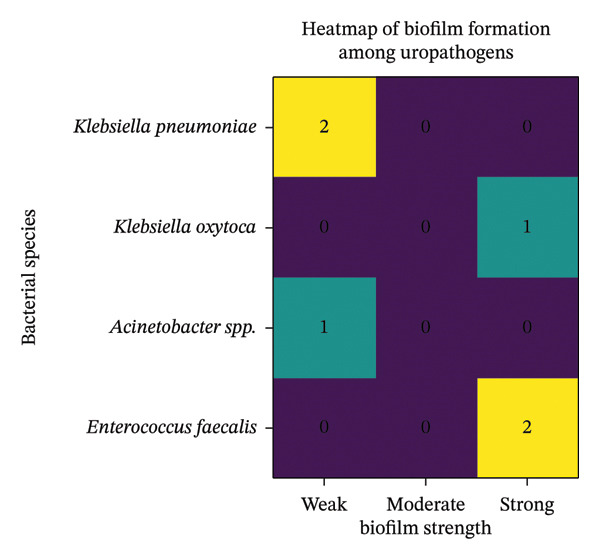
Heatmap of biofilm among uropathogens.

**FIGURE 4:**
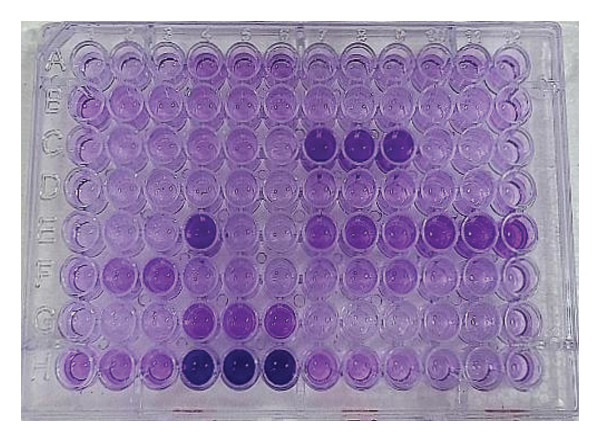
Microtiter plate biofilm assay. (A1, 2, 3 = blank, H4, 5, 6 = strong positive test).

Out of 184 bacterial isolates, biofilm production was observed only in *Klebsiella*, *Acinetobacter,* and *Enterococcus*. Six (3.26%) isolates were confirmed as biofilm producers, while the remaining 178 (96.7%) isolates were nonbiofilm producers (Figure 3). Figure 4 shows a microtiter plate used for biofilm production detection.

### 3.6. Correlation Between MDR and Biofilm Producers

In this study, six bacterial isolates were confirmed as biofilm producers, including MDR and non‐MDR strains. Among the strong biofilm producers, *K*. *oxytoca* was an MDR bacterium, while *E. faecalis* was non‐MDR. Furthermore, all weak biofilm‐producing isolates were MDR. The chi‐square test yielded a *p* value of 0.57, indicating no statistical significance between biofilm production and MDR status (Table 5).

**TABLE 5:** Relation between biofilm former and MDR.

MDR	Biofilm producer			
	Nonbiofilm producer	Weak	Moderate	Strong
MDR	32	1	0	1
Non‐MDR	152	2	0	2

## 4. Discussion

UTIs are the most common infections, and their treatment is challenging due to the emergence of MDR organisms. This issue is further aggravated by the easy formation of biofilms by microbes on both biotic and abiotic surfaces, resulting in higher levels of antibiotic resistance than their planktonic life. Biofilm‐associated UTIs are of significant concern in microbiology because, once established, these biofilms can serve as reservoirs for bacteria, leading to recurrent or chronic UTIs, as bacteria can persist despite treatment. If left untreated, these conditions can lead to numerous complications and ultimately decrease the quality of life [6, 8]. Our findings provide insights into the interplay between antibiotic resistance and biofilm formation in uropathogens, offering valuable insights for developing more effective strategies for preventing and controlling UTIs.

From a total of 2081 nonrepeated urine samples, the number of samples from females exceeded that from males. The age group of 20–40 years accounted for 41.32% of the samples, representing the highest number of patients with suspected UTIs. Significant bacteriuria was found in 184 clinical samples, indicating a point prevalence of UTI of 8.84%, which closely aligns with the global prevalence of 9.4% [14]. In contrast, this prevalence rate does not align with other studies in the Nepalese context, which reported prevalence rates ranging from 16% [15] to 20.3% [16], both based on sample sizes exceeding 1500 and conducted within similar time frames but at different tertiary care centers. This disparity may be due to variations in the population, increased screening practices, host factors, climatic conditions, socioeconomic status, educational level, hygiene practices, and other variables. The occurrence of UTIs was comparatively higher in females than in males, accounting for 57.61% and 42.39%, respectively. This higher frequency of UTIs in females is largely due to the anatomical and physiological differences between the sexes [10].

The incidence of UTIs was relatively consistent across the age groups of 20–40 and 40–60 years, with a slight decline observed in individuals over 60 years. Gram‐negative isolates were significantly more prevalent than Gram‐positive isolates, comprising 89.13% versus 10.87% of the isolates, respectively. Overall, the most common microbes were *E. coli* (39.7%), followed by *K. pneumoniae* (26.6%). The dominance of *E. coli* aligns with findings from other studies in Nepal, which reported varying frequencies of 59.8% from the College of Medical Sciences and Teaching Hospital, Chitwan [16], and 62.1% from Tribhuvan University Teaching Hospital [15]. However, it is noteworthy that the current frequency of *E. coli* appears to have decreased compared to earlier studies. The nonfermenting bacterium *P. aeruginosa,* although an unusual uropathogen, emerged as the third most common isolate in this study. *P. aeruginosa*, along with *Acinetobacter* spp., is primarily associated with healthcare‐associated infections [17]. Furthermore, the frequency of *P*. *aeruginosa* is more than that of *Acinetobacter*, as documented in other clinical settings [18, 19]. In our study, the prevalence of Gram‐positive isolates was less than 1% (0.96%) but accounted for 10.29% of the total isolates, which aligns with studies conducted in the eastern part of Nepal (11.15%) [20] and northern India (13.81%) [21]. Among these, *Enterococcus faecalis* was the most common, followed by coagulase‐negative *Staphylococcus* (CONS). This pattern is supported by studies from Spain [22] and a 10‐year retrospective study in Hungary [1].

Antibiotic treatment is essential for improving the quality of life of individuals with UTIs. However, the effectiveness of antibiotic intervention depends on the specific types of uropathogens involved and their antibiotic susceptibility patterns. The inappropriate practice of self‐medicating with antibiotics, without proper bacterial characterization, significantly contributes to the development and spread of antibiotic resistance. In our study, amikacin was the most effective antibiotic, whereas the efficacy of cotrimoxazole, a prototype drug frequently used for UTIs, decreased. For the predominant isolate, *E. coli*, amikacin showed the highest susceptibility, followed by nitrofurantoin, and amoxicillin was the least effective. The cephalosporin group of antibiotics, including cephalexin, ceftriaxone, and cefixime, showed resistance rates exceeding 20%. Compared to previous studies conducted in various regions of Nepal, India, and Pakistan [16, 20, 21, 23, 24], our findings indicated higher resistance rates for amikacin, amoxicillin, and cephalosporins. *Klebsiella* spp. was the second most common isolate, with amikacin being the most effective antibiotic for this pathogen. Notably, *K. pneumoniae* exhibited high resistance to several higher generation antibiotics, including ceftriaxone (40%), cefixime (49%), ceftazidime (92%), cefepime (100%), piperacillin (85%), piperacillin‐tazobactam (85%), imipenem, and meropenem (57%). A comparative analysis revealed that *K. oxytoca* showed a higher resistance rate to first‐line antibiotics than *K. pneumoniae,* but this trend was reversed for second‐line drugs for *K. pneumoniae*. Resistance to amikacin was found to be higher in a study conducted at Teaching Hospital [15] but lower in studies from other centers in Nepal, India, and Pakistan [16, 20, 21, 23, 24]. Further, our study revealed notably higher resistance rates to cefepime and piperacillin‐tazobactam for *K. pneumoniae*, which contrasts with findings from previous studies in Nepal and India, indicating a changing trend in susceptibility patterns [15, 20, 21, 23]. Similarly, the resistance rate for antibiotics targeting *Pseudomonas* was also high, and even the effective antibiotics amikacin and levofloxacin exhibited reduced susceptibility rates (28.6%). The antibiotic susceptibility test results for amikacin were consistent with those from a tertiary care center in Nepal [16]; however, the AST patterns for other antibiotics varied considerably.

For *Enterobacter* spp., tigecycline was the most effective antibiotic, whereas cephalosporins (ceftriaxone and cefixime), quinolones (levofloxacin and norfloxacin), amoxicillin‐clavulanic acid, and trimethoprim‐sulfamethoxazole were severely compromised. In case of *Citrobacter* spp., amikacin, levofloxacin, and norfloxacin showed complete susceptibility, whereas trimethoprim‐sulfamethoxazole and nitrofurantoin exhibited the highest resistance rates, each at 66.7%. For *Acinetobacter* spp., the least effective antibiotics were cefixime and ceftriaxone, both showing a resistance rate of 50%. For *Proteus mirabilis*, our study showed that amikacin, ceftriaxone, and cefixime were effective, exhibiting no resistance.

The antibiotic susceptibility pattern for CONS showed that 33.3% of isolates were resistant to both cefixime and trimethoprim‐sulfamethoxazole, while the remaining isolates remained susceptible. No antibiotic resistance was observed in *Enterococcus faecium.* In contrast, *E. faecalis* showed resistance rates of 25% to doxycycline and 60% to levofloxacin. Although resistance to nitrofurantoin, norfloxacin, and vancomycin among uropathogens was not reported in this study, emerging data suggest a rising prevalence of vancomycin‐resistant enterococci (VRE) among uropathogenic strains [25–27].

Increasing resistance to three or more classes of antibiotics has become a serious problem and poses a severe threat to the healthcare system worldwide. Both Gram‐negative and Gram‐positive organisms are developing resistance to antimicrobial therapy, resulting in higher morbidity and mortality rates. In this study, 17.4% (32) of the isolates were confirmed to be MDR. Among the MDR isolates, the highest number was observed in *K. pneumoniae* (14), followed by *P. aeruginosa* (9), *K. oxytoca* (5), and *E. coli* (2). Despite the overall higher number of *E. coli* isolates, the incidence of MDR was notably higher in *K. pneumoniae,* indicating that it is more frequently associated with MDR, which corroborates the findings of a study conducted in Guinea [28].

Biofilms are a prevalent mode of bacterial growth that significantly contribute to clinical infections. Infections associated with microbial biofilm tend to be recurrent, persistent, and challenging to treat, as the biofilm matrix protects bacteria from antimicrobial agents. Among the 184 isolates examined for biofilm production, only six (3.2%) were confirmed as biofilm producers. The relationship between MDR and biofilm formation remains unclear, with no clear evidence of a link between biofilm production and antimicrobial resistance [29, 30]. In our study, we analyzed the antibiotic resistance patterns and biofilm‐forming potential of bacterial isolates. Biofilm formation was observed in both MDR and non‐MDR strains. Among the biofilm producers, four isolates were MDR, and the remaining two, E. *faecalis*, isolates were non‐MDR. Overall, no significant correlation was found between antibiotic resistance and biofilm‐forming capacity of bacteria (*p* > 0.05), which aligns with previous studies [30, 31]. While many studies suggest that biofilms enhance bacterial survival, it cannot be conclusively stated that biofilm formation is always associated with antimicrobial resistance [32, 33]. The discrepancies in research findings may stem from variations in bacterial strains used for biofilm detection.

Further, there are various methods for detecting biofilm, among which the microtiter plate method serves as a valuable screening tool [34] with its certain limitations. The whole biomass staining with crystal violet indicates the lack of specificity, while expressing the result in absorbance value is relative rather than absolute. Additionally, results may vary from batch to batch due to differences in the washing and re‐solubilization processes. Furthermore, this method does not give the spatial organization of biofilm, i.e., ignoring in vivo dynamics in clinical conditions [35]. Despite its limitations, it can be addressed by detecting biofilm‐forming genes or counting colony‐forming units after biofilm disruption [34]. Additionally, confirming the presence or absence of antibiotic resistance genes clearly corroborates the relationship between biofilm formation and antibiotic resistance.

## 5. Conclusion

From this study, the majority of uropathogens in both sexes across all age groups were Gram‐negative isolates. The predominant isolate was *E. coli*, followed by *K. pneumoniae*. Multidrug resistance was higher in *K. pneumoniae* isolates, followed by *P. aeruginosa* and *K. oxytoca*. Biofilm formation was observed in both MDR and non‐MDR strains. In our study, *p* > 0.05, which suggested that there was no statistical correlation between antibiotic resistance and biofilm production by bacteria.

## Funding

The authors received no financial support for the research, authorship, or publication of this article.

## Ethics Statement

Ethical clearance was obtained from the Institutional Review Committee, Nobel College (Ref: 079/080/187).

## Consent

Written consent was obtained from each participant.

## Conflicts of Interest

The authors declare no conflicts of interest.

## Data Availability

The data will be available from the corresponding author upon reasonable request.
